# Acute Myocardial Infarction Due to Left Main Trunk Ostial Stenosis Occurring in the Acute Phase After Aortic Valve Replacement

**DOI:** 10.7759/cureus.47482

**Published:** 2023-10-22

**Authors:** Akiko Mano

**Affiliations:** 1 Cardiothoracic Surgery, Tokyo Metropolitan Institute for Geriatrics and Gerontology, Tokyo, JPN

**Keywords:** extracorporeal membrane oxygenation support, aortic complex geometry, aortic valve replacement, left main trunk, acute myocardial infarcation

## Abstract

Coronary ostial stenosis is a rare but critical complication after aortic valve replacement. We present a patient with acute myocardial infarction that occurred eight days after aortic valve replacement. The patient had favorable progress until eight days after the operation, but she suddenly developed ventricular fibrillation and then pulseless electrical activity; thus, she was placed on venoarterial extracorporeal membrane oxygenation. Emergent coronary angiography revealed severe stenosis without thrombus nor dissection in the left main trunk orifice, and we realized that the prosthetic valve stent was quite close to the left main trunk orifice. She underwent stent implantation, and TIMI III flow was achieved. She could be weaned from venoarterial extracorporeal membrane oxygenation in 12 days and was stabilized without inotropes. Unfortunately, she was complicated by fungal sepsis and died from multi-organ failure 37 days after index surgery. The majority of coronary ostial stenosis is reported to occur within a few months after surgery because of its pathophysiological mechanisms. The onset of coronary ostial stenosis in the acute phase after surgery like in our case is not common. The deformity of the aortic complex after aortic valve replacement may trigger a left main trunk ostial stenosis. The change of aortic complex geometry after aortic valve replacement should be noted, especially in small patients or narrow aortic annulus.

## Introduction

Coronary ostial stenosis (COS) after aortic valve replacement (AVR) is a rare but serious complication [1]. There are several speculated mechanisms for it, and one of the well-studied theories is micro-injuries during cardioplegia. Local hyperplastic reaction activated by injuries leads to stenosis and induces ischemia in a few months [2,3]. The symptoms are usually severe, and the patients need revascularization. We treated a patient with left main trunk (LMT) ostial stenosis followed by lethal arrhythmia and cardiogenic shock, in the acute phase, eight days after AVR. The coronary angiography (CAG) before AVR showed no significant stenosis. It was too early to develop intimal regeneration. We speculated that distortion related to valve replacement could cause LMT ostial stenosis. The change of aortic complex geometry after AVR should be noted, especially in small patients or narrow aortic annulus.

## Case presentation

**Figure 1:**
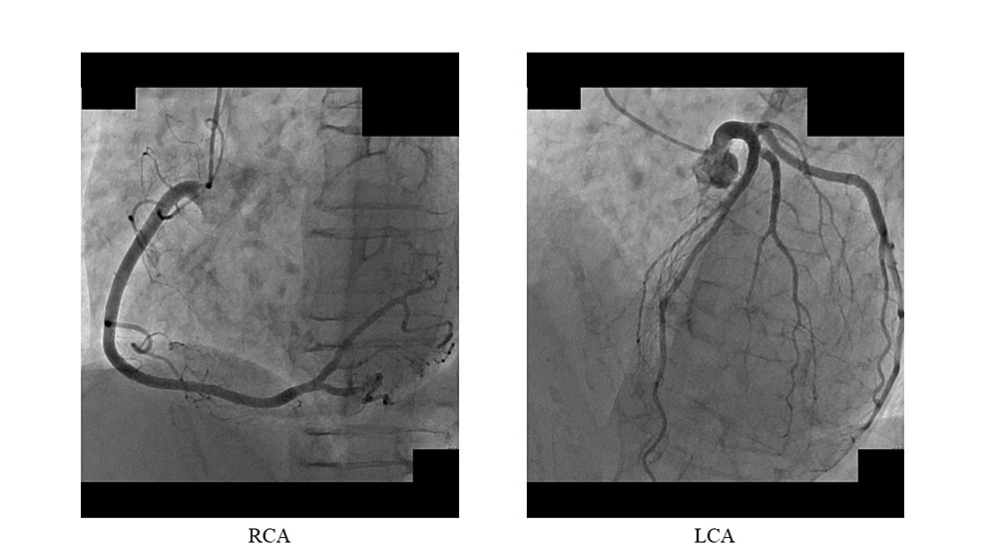
Preoperative CAG CAG, coronary angiography; RCA, right coronary artery; LCA, left coronary artery

A 71-year-old female with severe symptomatic aortic stenosis underwent AVR. She had a history of Parkinson’s disease for eight years. Her height, weight, and body surface area were 155 cm, 43 kg, and 1.4 m^2^, respectively. An echocardiogram before surgery showed no asynergy in the left ventricle (LV) with an ejection fraction (EF) of 75%. She had a bicuspid valve, and aortic valve peak velocity (APV) was 5.3 m/sec, peak/mean pressure (PPG/MPG) gradient was 114/69 mmHg, aortic valve area was 0.61 cm^2^, diameter of aortic annulus was 19x20 mm, and diameter of sinus Valsalva was 25 mm. A preoperative CAG showed no significant stenosis (Figure 1).

The aortic valve was replaced with a 21-mm Inspiris RESILIA valve (Edwards Lifesciences, Irvine, CA). The patient’s aortic valve is classified as a type 1 bicuspid aortic valve (right-left coronary cusp fusion) with complete raphe. We carefully removed the severely calcified bicuspid valve and placed 14 everted mattress sutures on the aortic annulus. One stent post of the biological valve was adjusted to the raphe position to avoid coronary occlusion. Other stent posts were not adjusted to the commissure position to avoid annular distortion. We sutured with 14 stitches using a braided suture accompanying 5-mm spaghetti-type pledgets (2-0 in diameter; Wayolax; Matsuda Ika Kogyo Company, Tokyo, Japan) via everting mattress. The bioprosthetic valve was implanted in the intra-annular position. The cardioplegic solution was intermittently administered by antegrade selective perfusion (initially 700 ml and then 500 ml every 30 minutes). The patency of bilateral coronary orifices was confirmed after valve replacement. Weaning from cardiopulmonary bypass was smooth, and the patient was transferred to an intensive care unit (ICU) under intubation with stable hemodynamics.

**Figure 2:**
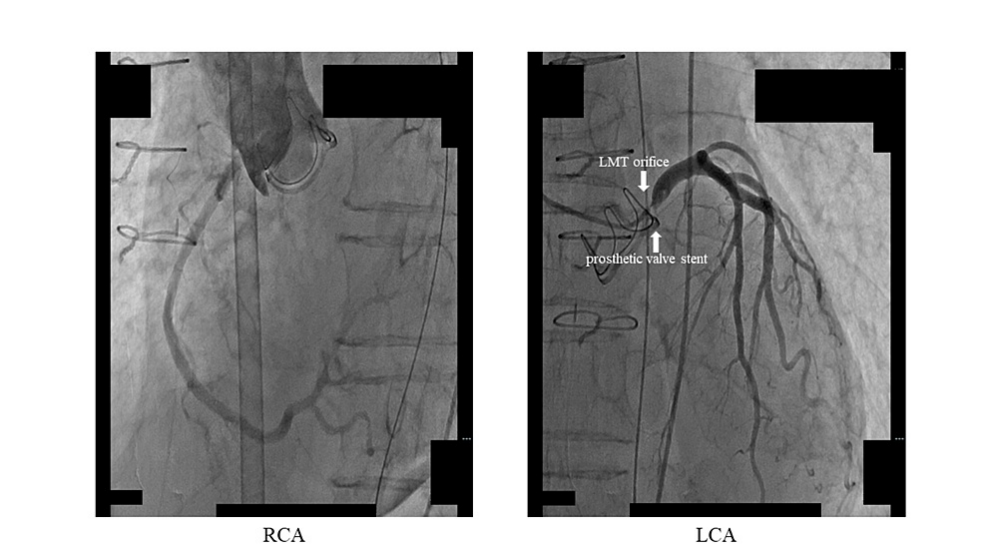
CAG eight days after AVR CAG, coronary angiography; AVR, aortic valve replacement; RCA, right coronary artery; LCA, left coronary artery; LMT, left main trunk

The clinical course after surgery was uneventful. She was extubated five hours after ICU admission and was weaned off inotropes on postoperative day (POD) 1 and moved to a general ward on POD 2. Until POD 8, she had been doing very well. An echocardiogram on POD 7 showed good LV wall motion with an EF of 71%. A bioprosthetic valve function was fine, with an APV of 1.54 m/sec, and PPG/PMG of 9.5/4.7 mmHg, without perivalvular or transvalvular regurgitations. Her PT-INR at that time was 2.22 on Coumadin, which was started on POD 3. However, she suddenly developed ventricular fibrillation on POD 8. We immediately performed cardiopulmonary resuscitation, but she fell into pulseless electrical activity; therefore, we emergently placed her on venoarterial membrane oxygenation (VA-ECMO). She returned to spontaneous circulation on VA-ECMO. The resuscitation time was about 20 minutes. After the return of spontaneous circulation, an electrocardiogram revealed an ST-segment elevation in leads V1-6 and I aVL, and an echocardiogram showed akinesis of the LV wall except for the inferior wall with an EF of 10%. She was diagnosed with LMT ischemia and got emergent CAG. The CAG demonstrated a 99% stenosis in the LMT orifice without thrombus nor obvious dissection (Figure 2).

**Figure 3:**
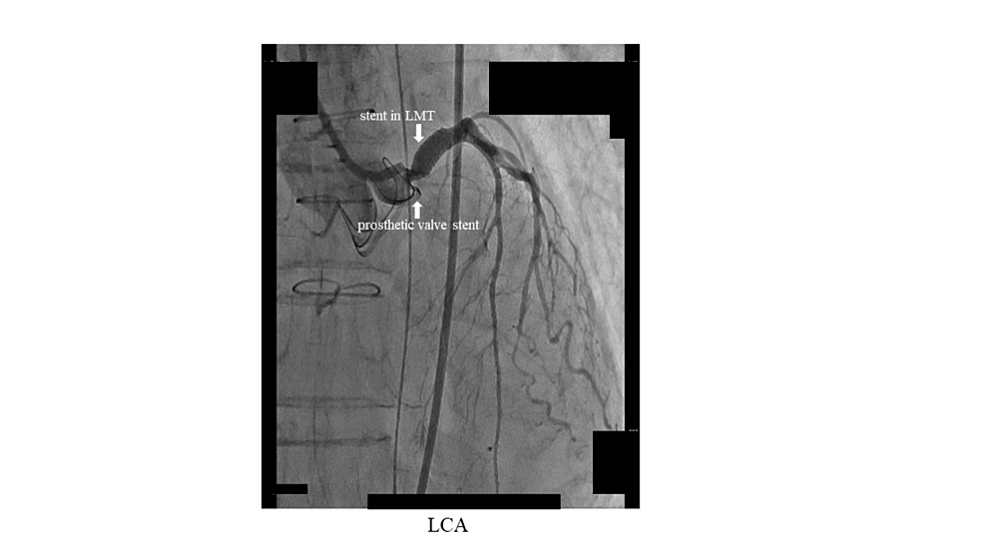
CAG after stent implantation in LMT CAG, coronary angiography; LCA, left coronary artery; LMT, left main trunk

She subsequently underwent stent (4.0x16 mm; SYNERGY, Boston Scientific, Marlborough, MA) implantation and got TIMI grade 3 flow (Figure 3).

**Figure 4:**
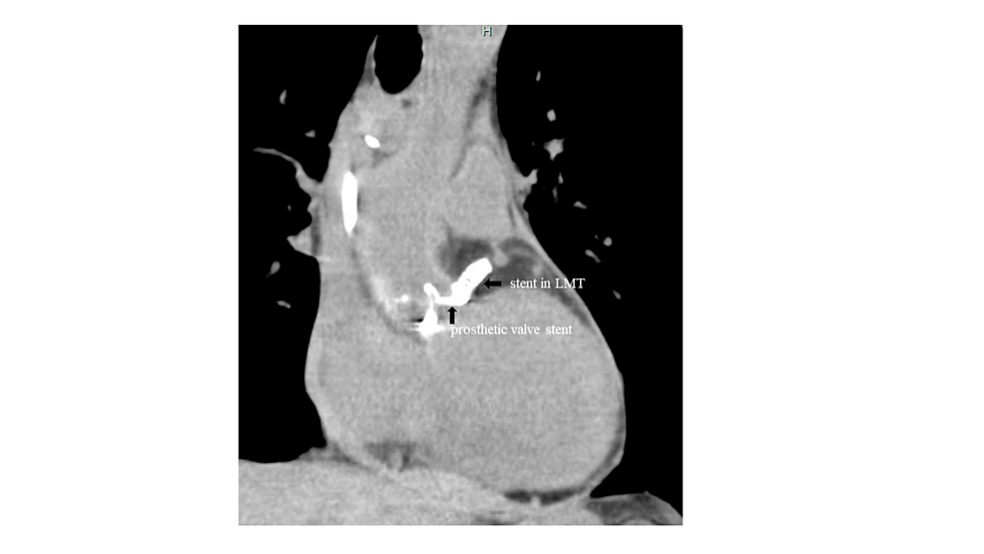
Chest computed tomography after stent implantation in LMT LMT, left main trunk

We carefully placed the stent not to extend to the ascending aorta for possible transcatheter aortic valve replacement in the future. After stent implantation, we realized that the prosthetic valve stent was quite close to the LMT orifice (Figures 2, 3). The same finding was observed by computed tomography after stent implantation, which revealed that a stent in LMT was almost in contact with a prosthetic valve (Figure 4).

Her maximum level of creatinine kinase (CK) was 13,300 IU/L eight hours after percutaneous coronary intervention (PCI). She had frequent episodes of ventricular tachycardia for a few days that responded to medical treatment. Her neurological function was good, and her hemodynamic status gradually stabilized; thus, we started to reduce VA-ECMO flow and successfully weaned it off on the 12th day after PCI. An echocardiogram on the 18th day after PCI showed broad asynergy, akinesis in the apex, anteroseptal to lateral wall, and low LV function with an EF of 25%, but her hemodynamic status was maintained without inotropes. Her general condition slowly improved. Unfortunately, she was complicated by fungal sepsis on the 21st day after PCI and developed shock and multi-organ failure, and she finally died on the 29th day after PCI (on the 37th day after AVR).

## Discussion

COS is recognized as a rare but serious complication after AVR [1]. It is reported that its incidence is 1%-5%, and clinical symptoms are usually severe such as broad myocardial ischemia, life-threatening arrhythmias, congestive heart failure, or sudden cardiac death [2-4]. COS occurs on both sides, but it is more frequently seen in the left coronary artery [3].

Several mechanisms regarding COS are investigated. The possibility of microinjuries and local hyperplastic reactions related to the infusion pressure of the cardioplegic fluid and over-dilatation of vessels by the tip of a cardioplegic catheter is one of the most well-described mechanisms [2-4]. The turbulence flow around the prosthetic valve may provoke intimal thickening and fibrous proliferation in the aortic root and proximal coronary artery that leads to COS [1,5]. The exposure of direct blood flow from the left ventricle to LMT by removing native aortic cusps could result in ostial injuries, and it induces stenosis [6]. An immunologic reaction, the deposition of circulating immune complexes on the vascular wall, might cause stenosis [7]. Because of these pathophysiological mechanisms, COS is usually seen in a few months after AVR [2,4].

In our patient, LMT ostial stenosis occurred in the acute phase, about one week after AVR. That is uncommon. CAG before AVR showed normal coronary arteries. It is too early to cause coronary ostial degeneration reported before. Therefore, we speculated that a different mechanism would be related to our case. Our patient was relatively small, so the diameter of the sinus of Valsalva and aortic annulus tended to be narrow as previously mentioned. Thus, the aortic complex might change shape after valve replacement causing twitching around the LMT orifice and induced stenosis. Actually, CAG after AVR revealed that the prosthetic valve stent was quite close to the LMT orifice (Figures 2, 3). The same finding was observed by computed tomography after PCI, which showed that a stent in LMT was almost in contact with the prosthetic valve (Figure 4).

Farid et al. found that 11 out of 7,507 AVR patients died from coronary ostial stenosis/occlusion, and most of them got a 21 mm valve, although the most common valve size was 23 mm. They suggested that a small aortic root is a risk factor for COS [8]. This finding is consistent with our speculation. An echocardiogram on the day before AMI showed no asynergy in LV wall motion with an EF of 71% and good prosthetic valve function. We thought that if an aortic complex deformity existed just after AVR, then hypovolemia and blood pressure fluctuation because of Parkinson’s disease in addition to LMT ostial stenosis could induce low perfusion pressure and cause AMI. Actually, her body weight decreased by 1.6 kg on POD 8 compared with it before surgery, and her systolic blood pressure ranged from 90 mmHg to 150 mmHg after surgery.

There are a few reports about COS in the acute phase after AVR. They showed that ostial thrombus from aortic retractor trauma, coronary artery spasm, and calcific embolism might result in ostial stenosis [4]. Ostial dissection by cardioplegic cannula could lead to stenosis both in the acute and the chronic phase after AVR, which could be avoided by modifying cardioplegic methods, such as retrograde perfusion or single-dose cardioplegia [9,10]. However, they were inconsistent with our case based on CAG findings.

PCI for COS after AVR has recently increased, although most COS have previously needed coronary artery bypass grafting. We successfully treated LMT ostial stenosis by PCI, and we could stabilize hemodynamic status and wean VA-ECMO off. We could have saved the patient if she had not been complicated by sepsis.

## Conclusions

We experienced a patient with AMI because of LMT ostial stenosis eight days after AVR. The change of aortic complex geometry after valve replacement could induce this complication. Patients with smaller aortic complexes would be at higher risk of COS after AVR, and we should be careful about those patients.
